# Impact of a diverting ileostomy in total mesorectal excision with primary anastomosis for rectal cancer

**DOI:** 10.1007/s00464-022-09669-x

**Published:** 2022-10-18

**Authors:** Jeroen C. Hol, Thijs A. Burghgraef, Marieke L. W. Rutgers, Rogier M. P. H. Crolla, Anna A. W. van Geloven, Gabie M. de Jong, Roel Hompes, Jeroen W. A. Leijtens, Fatih Polat, Apollo Pronk, Anke B. Smits, Jurriaan B. Tuynman, Emiel G. G. Verdaasdonk, Esther C. J. Consten, Colin Sietses

**Affiliations:** 1 Department of Surgery, Amsterdam University Medical Center, Location VU Medical Centre, De Boelelaan 117, 1081 HB Amsterdam, The Netherlands; 2 Department of Surgery, Hospital Gelderse Vallei, Ede, The Netherlands; 3 Department of Surgery, Meander Medical Centre, Amersfoort, The Netherlands; 4 Department of Surgery, University Medical Centre Groningen, Groningen, The Netherlands; 5 Department of Surgery, Amsterdam University Medical Center, Location Academic Medical Centre, Amsterdam, The Netherlands; 6 Department of Surgery, Amphia Hospital, Breda, The Netherlands; 7 Department of Surgery, Tergooi Hospital, Hilversum, The Netherlands; 8 Department of Surgery, Laurentius Hospital, Roermond, The Netherlands; 9 Department of Surgery, Canisius Wilhelmina Hospital, Nijmegen, The Netherlands; 10 Department of Surgery, Diakonessenhuis, Utrecht, The Netherlands; 11 Department of Surgery, St. Antonius Hospital, Nieuwegein, The Netherlands; 12 Department of Surgery, Jeroen Bosch Hospital, Den Bosch, The Netherlands

**Keywords:** Ileostomy, Laparoscopy, Rectal cancer

## Abstract

**Background:**

The role of diverting ileostomy in total mesorectal excision (TME) for rectal cancer with primary anastomosis is debated. The aim of this study is to gain insight in the clinical consequences of a diverting ileostomy, with respect to stoma rate at one year and stoma-related morbidity.

**Methods:**

Patients undergoing TME with primary anastomosis for rectal cancer between 2015 and 2017 in eleven participating hospitals were included. Retrospectively, two groups were compared: patients with or without diverting ileostomy construction during primary surgery. Primary endpoint was stoma rate at one year. Secondary endpoints were severity and rate of anastomotic leakage, overall morbidity rate within thirty days and stoma (reversal) related morbidity.

**Results:**

In 353 out of 595 patients (59.3%) a diverting ileostomy was constructed during primary surgery. Stoma rate at one year was 9.9% in the non-ileostomy group and 18.7% in the ileostomy group (*p *= 0.003). After correction for confounders, multivariate analysis showed that the construction of a diverting ileostomy during primary surgery was an independent risk factor for stoma at one year (OR 2.563 (95%CI 1.424–4.611), *p *= 0.002). Anastomotic leakage rate was 17.8% in the non-ileostomy group and 17.2% in the ileostomy group (*p *= 0.913). Overall 30-days morbidity rate was 37.6% in the non-ileostomy group and 56.1% in the ileostomy group (*p *< 0.001). Stoma reversal related morbidity rate was 17.9%.

**Conclusions:**

The stoma rate at one year was higher in patients with ileostomy construction during primary surgery. The incidence and severity of anastomotic leakage were not reduced by construction of an ileostomy. The morbidity related to the presence and reversal of a diverting ileostomy was substantial.

**Supplementary Information:**

The online version contains supplementary material available at 10.1007/s00464-022-09669-x.

Total mesorectal excision (TME), often combined with neoadjuvant treatment is standard of care for curative rectal cancer treatment [1, 2]. The introduction of minimally invasive techniques reduced morbidity, infection rates and length of postoperative hospital stay [3, 4]. When possible, a sphincter-saving procedure is performed with an anastomosis to regain bowel continuity after resection.

Anastomotic leakage after a sphincter-saving procedure is a serious complication associated with severe morbidity [5]. It is a common complication, with an incidence up to 20% [5, 6]. Moreover, it predisposes rectal cancer patients to worse oncological outcomes [7]. Treatment of anastomotic leakage can result in anastomotic take-down with permanent stoma rates of 20%, associated with a significant impact on quality of life [8].

Construction of a temporary loop ileostomy during sphincter-saving TME surgery is a well-known procedure. A diverting stoma does not decrease the risk of anastomotic leakage. However, it might reduce clinical anastomotic leakage and reoperation rates [9, 10]. As a disadvantage a diverting ileostomy itself can induce significant discomfort, morbidity and impact on quality of life [8, 11, 12]. Stoma-related complications such as dermatitis, stoma dysfunction or high output stoma occur in more than half of the cases and result in more hospital admissions [11]. Moreover, patients have to go through a second surgery for stoma closure, which is associated with significant risks and morbidity as well [11]. All these stoma-related issues are associated with increased treatment costs [13].

Taking the above into account, routine diversion is increasingly debated. Already, there seems to be a large variation in the selection of patients who will receive a diverting stoma where surgeons’ preference and patient- and tumour related factors seem to play a role [14]. A temporary loop ileostomy is constructed in 76% of patients undergoing a TME, varying from 0 to 100% between centres [14]. Finally, a significant proportion of the diverting stomas are never closed [15]. Sometimes a secondary stoma is constructed after reversal and construction of a diverting ileostomy might even increase the risk of a permanent stoma [16, 17].

Most previous studies focussed on the impact of diversion on anastomotic leakage and several studies have assessed the efficacy of high selective diversion only, instead of routine diversion [18–20]. Unfortunately, only a few studies concentrated on the high numbers of stoma-related complications and the risk of a permanent stoma after loop ileostomy construction [8, 11, 12]. Therefore, the aim of this study is to gain in depth insight in the clinical consequences of a diverting ileostomy after TME with primary anastomosis for rectal cancer with respect to stoma rate at one year and stoma-related morbidity.

## Materials and methods

### Study design and patients

A retrospective multicentre cohort study was performed in eleven hospitals in the Netherlands. A study protocol was composed prior to initiation of the study and approved by the MEC-U medical ethics committee (AW 9.023/W18.100) and by the local boards of all participating hospitals.

All patients of 18 years old or older, diagnosed with histologically proven rectal cancer and operated between January 2015 and December 2017 were included. Excluded from analysis were patients without construction of a primary anastomosis, with sigmoidal tumours according to the sigmoidal take-off definition [21], with recurrent rectal cancer, with presence of multiple colonic tumours, that underwent transanal endoscopic microsurgery (TEM) or with construction of an end colostomy. Neoadjuvant treatment was administered, when deemed necessary according to the Dutch national guidelines [22]. No adjuvant therapy was administered, according to the Dutch guidelines. Each of the eleven participating hospitals performed at least 40 procedures per year, performing either laparoscopic, robot-assisted or transanal TME. Construction of a diverting ileostomy was based on the attending surgeon’s choice. All patients were treated according to local multidisciplinary enhanced recovery after surgery (ERAS) protocols, when possible. Stoma reversal was planned within a few months after primary surgery, based upon the hospitals’ local protocol. All patients had follow-up carried out according to the Dutch National Guidelines for Colorectal Cancer for a period of 5 years.

Data were derived from the Dutch Colo Rectal Audit (DCRA) [23]. Data not captured in this nationwide audit were completed using the local electronic medical record (EMR). Any missing variables were added to the database by one of the researchers using the EMR, including: location of the tumour on MRI, details on type of operation, anastomosis, intra-operative complications, postoperative complications, stoma-related complications and details on stoma reversal and reversal related complications. Patients were pseudo anonymised before consulting the EMR for data collection. All data were collected between January and April 2020 and stored in the data management system CASTOR.

A comparison was made between patients with or without diverting ileostomy construction during primary surgery. Subgroup analysis was performed for patients with anastomotic leakage. Univariate logistic regression followed by multivariate logistic regression with respect to patient and tumour related factors was performed for morbidity rates within 30 days, anastomotic leakage rates and stoma rate at one year postoperatively.

### Outcomes and definitions

Baseline characteristics included were: age, sex, body mass index (BMI), ASA classification (American Society of Anesthesiologists), tumour height from the anorectal junction (ARJ) in centimetres based on pre-treatment MRI, tumour height based on pre-treatment MRI according to criteria from “The English National Low Rectal Cancer Development Programme”(LOREC) [24], clinical TNM staging based on MRI, mesorectal fascia (MRF) involvement on MRI, administration of pre-operative (chemo)radiation therapy, type of surgery, intra-operative details on stapled or hand sewn anastomosis, presence of intra-operative complications, conversion to laparotomy and operating time in minutes. Length of initial hospital stay was defined as the number of postoperative days during initial admission. Complications related to primary surgery were categorised according to Clavien-Dindo [25]. All reinterventions and readmissions within 30 days were scored. Stoma-related complications after 30 days were scored if they required any readmission.

Anastomotic leakage was defined as anastomotic dehiscence or intra-abdominal abscess adjacent to the anastomotic site, requiring radiological or surgical intervention during follow-up. Anastomotic leakage was graded according to the need for intervention, based on the definition of the International Study Group of Rectal Cancer (ISGRC) [26]. Grade A requires no change of management, grade B requires intervention other than relaparotomy and grade C requires relaparotomy. A secondary ileostomy was defined as a stoma constructed during a second procedure.

Primary endpoint was the overall stoma rate at one year, which included the presence of any type of stoma one year after primary surgery. Secondary endpoints were the overall morbidity rate within 30 days, the rate of anastomotic leakage and stoma reversal related morbidity.

### Statistical analysis

Data of categorical variables were presented as numbers (%). Data of continuous variables were presented as mean (standard deviation) or median [interquartile range] depending on the type of distribution. Comparison of categorical data was done using a Chi-square test, or Fishers exact test. Comparison of continuous data between groups was done using a T-test in case of a normal distribution or Mann–Whitney-U test in case of a non-normal distribution. After univariate logistic regression, multivariate logistic regression was performed using backward selection. For anastomotic leakage grade C propensity score adjusted multivariate regression was performed because of low incidence of the primary outcome, and subsequent suspected problems with overfitting. For anastomotic leakage rate and complications within 30 days rate, univariate analysis was performed for sex, age, BMI, ASA, distance from ARJ on MRI, neoadjuvant treatment, conversion and intra-operative complications. For stoma rate at one-year follow-up, univariate analysis was performed for sex, age, BMI, ASA, distance from ARK on MRI, neoadjuvant treatment, conversion, intra-operative complications, cTNM stage and anastomotic leakage. All statistical analyses were carried out using SPSS Statistics version 24 (IBM, Chicago, IL, USA).

## Results

**Fig. 1:**
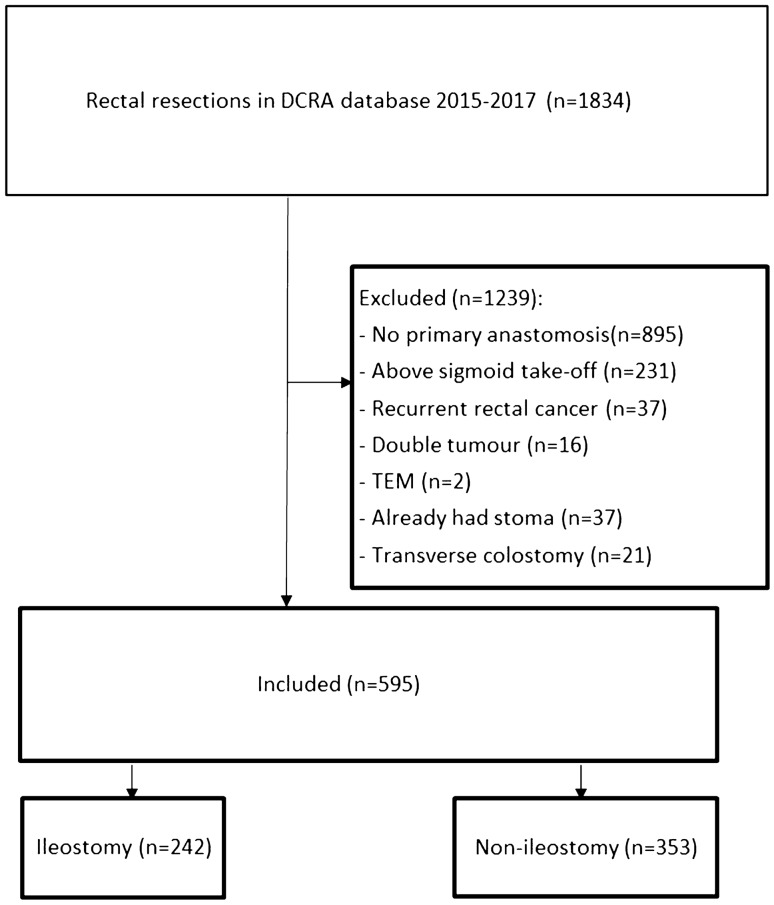
Flowchart

A total of 1834 patients were registered in the DCRA between 2015 and 2017 in the participating hospitals. A total of 595 underwent sphincter-saving TME surgery for rectal cancer and met the inclusion criteria. In 353 patients (59.3%) a diverting ileostomy was constructed at primary surgery. An overview can be seen in the flow diagram (Fig. 1). The hospitals’ unadjusted proportion of diverting ileostomy construction varied from 7.1 to 83.0% (supplementary Fig. 1).

### Characteristics

Comparing the ileostomy group to the non-ileostomy group, the ileostomy group had more male patients in the ileostomy group (68.3% vs 56.2%, *p *= 0.003), more MRI-defined low rectal cancers (43.9% vs 36.8%, *p *= 0.010), more cT3-4 tumours (*p *< 0.001), more neoadjuvant (chemo)radiation therapy administered (75.6% vs 38.4%, *p *< 0.001), and less cN0 stage (34.3% vs 61.6%, *p *< 0.001). Median length of follow-up was longer in the ileostomy group than in the non-ileostomy groups (38[46–48] vs 36[24–45] months, *p *= 0.019). Table 1 provides an overview of all characteristics of both groups.

**Table 1:** Characteristics

	Non-ileostomy		Ileostomy		Total		*P* value
	*N* = 242	%	*N* = 353	%	*N* = 595	%	
Sex							
Male	136	56.2	241	68.3	377	63.4	0.003
Female	106	43.8	112	31.7	218	36.6	
Age (years)							
Mean(SD)	64.5(10.0)		63.8(9.3)		64.0(9.6)		0.397
BMI							
Mean(SD)	25.7	4.2	26.1	3.8	25.9	3.98	0.287
ASA							
I	62	25.6	85	24.1	147	24.7	0.377
II	139	57.4	223	63.2	362	60.8	
III	39	16.1	44	12.5	83	13.9	
IV	2	0.8	1	0.3	3	0.5	
Height from ARJ on MRI (cm)							
Median[IQR]	7[5–9]		6[4.5–8]		6.5	[4.5–9.0]	0.002
MRI-defined LOREC low rectal cancer							
Yes	89	36.8	155	43.9	244	41.0	0.010
Clinical tumour stage							
T1	12	5.0	10	2.8	22	3.7	< 0.001
T2	104	43.0	63	17.8	167	28.1	*
T3	119	49.2	255	72.2	374	62.9	*
T4	7	2.9	25	7.1	32	5.4	*
Clinical Nodal stage							
N0	149	61.6	121	34.3	270	45.4	0.000*
N1	74	30.6	125	35.4	199	33.4	
N2	18	7.4	107	30.3	125	21.0	*
Unknown	1	0.4	0	0,0	1	0.2	
Synchronous metastasis							
Yes	12	5.0	34	9.6	46	7.7	0.111
Preoperative therapy							
No	149	61.6	86	24.4	235	39.5	< 0.001
(chemo)radiation	93	38.4	267	75.6	360	60.5	
Type of surgery							
Open	5	2.1	7	2.0	12	2.0	0.027
laparoscopic	98	40.5	122	34.6	220	37.0	
TaTME	77	31.8	92	26.1	169	28.4	
Robotic	62	25.6	132	37.4	194	32.6	*
Technique of anastomosis							
Handsewn	7	2.9	14	4.0	21	3.5	0.753
Stapled	234	96.7	336	95.5	570	96.0	
Robotic stapler	1	0.4	2	0.6	3	0.5	
Type of anastomosis							
Side to side	59	24.4	82	23.3	141	23.7	0.456
End to side	134	55.4	215	61.1	349	58.8	
End to end	41	16.9	46	13.1	87	14.6	
Other configuration	8	3.3	9	2.6	17	2.9	
Intra-operative complications							
Yes	13	5.4	18	5.1	31	5.2	1.000
Duration of operation minutes							
Mean(SD)	184.3(83.2)		194.9(67.3)		190.5(74.3)		0.086
Conversion to laparotomy							
Yes	6	2.5	15	4.2	21	3.5	0.270
Length of follow-up in months							
Median[IQR]	36[24–45]		38[26–48]		37[26–47]		0.019

Numbers in parentheses are percentages, unless mentioned otherwise

*BMI* Body Mass Index (kg/m^2^), *SD* standard deviation, *ASA* American Society of Anesthesiologists, *cm* centimeters, *ARJ* anorectal junction, *LOREC* MRI-defined low rectal cancer below insertion of levator muscle, *IQR* interquartile range

^*^Post hoc test significant for this category

### Thirty-day morbidity

Table 2 shows an overview of the morbidity within 30 days postoperatively in both groups. Thirty-day morbidity rates were significantly higher in the ileostomy group than in the non-ileostomy group (56.1% vs 37.6%, *p *< 0.001). This was confirmed in a multivariate logistic regression analysis, after correction for sex and tumour distance from ARJ (OR 2.037(95%CI 1.434–2.892), *p *< 0.001), see Supplementary Table 1. Severe complications (Clavien-Dindo grade III or higher) were less frequently seen in the ileostomy group (39.7 vs 61.5%, *p *= 0.001) and median days of ICU admission was shorter in the ileostomy group (1[1, 2] vs 1[1], *p *= 0.046). The overall surgical complication rate was higher in the ileostomy group (42.8% vs 27.7%, *p *< 0.001), with the presence of ileus having the highest incidence in the ileostomy group (24.1% vs 8.3%, *p *< 0.001). Moreover, more readmissions within 30 days occurred in the ileostomy group (20.1% vs 11.2%, *p *= 0.003) and median length of hospital stay in days was longer (7[5–15] vs 5[4–7], *p *< 0.001).

**Table 2:** Morbidity

	Non-ileostomy		Ileostomy		Total		*p*-value
	*N* = 242	%	*N* = 353	%	*N* = 595	%	
Complications within 30 days	91	37.6	198	56.1	289	48.6	< 0.001
Pulmonary complications	9	3.7	21	5.9	30	5.0	0.256
Most severe complication (Clavien-Dindo)**							
Mild Grade 1–2	35	38.5	120	60.3	155	53.4	0.001
Severe Grade 3 or higher	56	61.5	79	39.7	13.5	46.6	
Cardiac complications	8	3.3	13	3.7	21	3.5	0.827
Thrombotic event	2	0.8	8	2.3	10	1.7	0.213
Infection not pulmonary or surgical	16	6.6	34	9.6	50	8.4	0.229
Neurological	6	2.5	10	2.8	16	2.7	0.806
Urological	19	7.9	20	5.7	39	6.6	0.314
Other	10	4.1	41	11.6	51	8.6	0.001
Surgical complications within 30 days	67	27.7	151	42.8	218	36.6	< 0.001
Abscess	10	4.1	7	2.0	17	2.9	0.138
Bleeding	8	3.3	5	1.4	13	2.2	0.155
Ileus	20	8.3	85	24.1	105	17.6	< 0.001
Fascia dehiscence	1	0.4	3	0.8	4	0.7	0.650
Bowel perforation	3	1.2	2	0.6	5	0.8	0.653
Ureter/bladder leak	1	0.4	1	0.3	2	0.3	0.788
Wound infection	7	2.9	10	2.8	17	2.9	0.966
Other	9	3.7	26	7.4	35	5.9	0.076
Anastomotic leak (any)							
Yes	43	17.8	61	17.3	104	17.5	0.913
Reinterventions within 30 days							
Yes	52	21.5	71	20.1	123	20.7	0.757
Readmission within 30 days							
Yes	27	11.2	71	20.1	98	16.5	0.003
Number of readmissions							
1	23	85.2	58	81.7	81	82.7	0.896
2	3	11.1	10	14.1	13	13.3	
3	1	3.7	3	4.2	4	4.1	
Reason for readmission							
Anastomotic leakage or abscess	14	51.9	25	35.2	39	39.8	0.014
Ileus	0	0.0	15	21.1	15	15.3	*
Obstipation	1	3.7	1	1.4	2	2.0	
Stoma-related	1	3.7	13	18.3	14	14.3	
Infection not pulmonary or surgical	2	7.4	1	1.4	3	3.1	
Other type	9	33.3	16	22.5	25	25.5	
Total days of readmission within 30 days							
Median [IQR]	11[3–35.5]		5.5[2.25–14.0]		5.5[3.0–13.0]		0.744
Length of hospital stay (days)							
Median[IQR]	5[4–7]		7[5–15]		6[4–12]		< 0.001
Days of ICU admission							
Median[IQR]	1[1, 2]		1[1–1]		1[1–1]		0.046

Numbers in parentheses are percentages. Unless mentioned otherwise

*IQR* interquartile range

^*^Post hoc test significant for this category

^**^Of 290 patients with complications

### Stoma-related morbidity

Table 3 shows an overview of the stoma-related morbidity in both groups. In the non-ileostomy group 43 patients (17.8%) had secondary ileostomy construction. At four weeks postoperatively, 96.6% had a stoma in the ileostomy group and 15.3% of patients had a stoma in the non-ileostomy group (*p *< 0.001). The rate of stoma-related complications within 30 days was 45.5% in the ileostomy group and 6.6% in the non-ileostomy group (*p *< 0.001). Stoma-related complications during the remaining follow-up were 20.5% in the ileostomy group, 3.4% of them underwent stoma-related interventions**.** In the non-ileostomy group 17.8% had a secondary stoma at any time point during the first year.

**Table 3:** Stoma-related morbidity

	Non-ileostomy		Ileostomy		Total		*p*-value
	*N* = 242	%	*N* = 353	%	*N* = 595	%	
Stoma-related morbidity within 30 days	6	6.6	90	45.5	96	33.2	< 0.001
High output/dehydration	3	1.2	67	19.0	70	11.8	< 0.001
Prolapse	1	0.4	1	0.3	2	0.3	1.000
Parastomal hernia	0	0.0	3	0.8	3	0.5	0.275
Other	4	1.7	25	7.1	29	4.9	0.003
Had stoma during first year							
Constructed at primary resection	0	0.0	333	94.3	333	56.0	< 0.001*
After primary resection because of complication	40	16.5	7	2.0	47	7.9	*
After reversal new stoma	0	0.0	9	2.5	9	2.5	*
Other	3	1.2	4	1.1	7	1.2	
Never had a stoma	199	82.2	0	0.0	199	33.4	*
Presence of a stoma at 1 year	24	9.9	66	18.7	90	15.1	0.003
Type of stoma 1 year							
Stoma free	218	90.1	287	81.3	505	84.9	0.013*
Loop ileostomy	12	5.0	48	13.6	60	10.1	*
End ileostomy	0	0.0	1	0.3	1	0.2	
Loop colostomy	1	0.4	2	0.6	3	0.5	
End colostomy	11	4.5	15	4.2	26	4.4	
Stoma-related complications after 30 days	16		72	20.5	88	21.9	0.101
Ileus	0	0.0	5	1.4	5	0.8	0.084
Prolapse	3	1.2	6	1.7	9	1.5	0.745
Parastomal hernia	5	2.1	7	2.0	12	2.0	1.000
Stricture	1	0.4	2	0.6	3	0.5	1.000
Dehiscence	0	0.0	1	0.3	1	0.2	1.000
Necrosis	1	0.4	1	0.3	2	0.3	1.000
Skin issues	6	2.5	45	12.7	51	8.6	< 0.001
High output/dehydration	4	1.7	14	4.0	18	3.0	0.144
Other	1	0.4	3	0.8	4	0.7	0.650
Stoma-related reinterventions during follow-up	5	2.1	12	3.4	17	2.8	0.044
Number of stoma-related reinverventions							
Median[IQR]	1[1]		1[1]		1[1]		0.799
Stoma-related readmissions after 30 days**	0	0.0	8	2.3	8.0	1.3	
Number of stoma-related readmissions							
1	0	0.0	7	87.5	7.0	87.5	
3	0	0.0	1	12.5	1.0	12.5	
Total days of stoma-related readmissions after 30 days							
Median [IQR]	NA		6[2.25–10.5]		6[2.25–10.5]		
Reason non reversal							
Patient preference	1	4.8	3	10.3	4	8.0	0.415
Palliative treatment	3	14.3	7	24.1	10	20.0	
Expected poor functional outcome	3	14.3	3	10.3	6	12.0	
Underwent APR	4	19.0	1	3.4	5	10.0	
Other	10	47.6	15	51.7	25	50.0	

Numbers in parentheses are percentages. Unless mentioned otherwise

*IQR* interquartile range, *APR* abdominoperineal resection

^*^Post hoc test significant for this category

^**^5 had high output ileostomy, 1 had parastomal hernia, 2 had ileus

### Stoma rate at one year

**Fig. 2:**
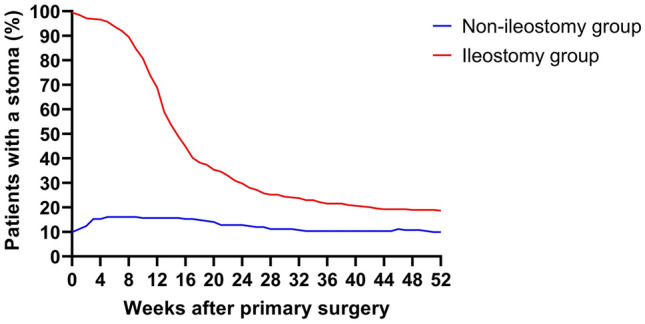
Presence of a stoma during one-year follow-up Non-ileostomy group, Ileostomy group

At one year postoperatively, 18.7% of the patients in the ileostomy group and 9.9% of the patients in the non-ileostomy group had a stoma (*p *= 0.003). This difference in stoma rate at one year was maintained after a multivariate logistic regression analysis with correction for sex, age and anastomotic leakage (OR 2.563 (95%CI 1.424–4.611), *p *= 0.002), as can be seen in supplementary Table 1. Figure 2 shows the presence of stoma during one year follow-up in both groups.

### Anastomotic leakage

The overall rate of anastomotic leakage did not differ between groups (17.3% in the ileostomy group and 17.8% in the non-ileostomy group, *p *= 0.913), see Table 2. Table 4 gives an overview of a subgroup analysis of the morbidity after anastomotic leakage in 104 patients. The rate of grade B leakage was higher in the ileostomy group than in the non-ileostomy group (49.2% vs 14%, *p *< 0.001). Grade C leakage rate was lower in the ileostomy group compared to the non-ileostomy group (29.5% and 76.7% of all leakages, respectively, *p *< 0.001). This was confirmed by a multivariate analysis after correction for sex, tumour distance from ARJ and neoadjuvant therapy [OR 0.263 (95%CI 0.138–0.505), *p *< 0.001], see supplementary Table 1. In all patient in the non-ileostomy group with a grade C leakage a stoma was constructed during reoperation. In 25 out of 33 (75.8%) of these patients an ileostomy was constructed. The others required direct take-down of the anastomosis.

**Table 4:** Subgroup analysis of anastomotic leakage

	Non-ileostomy		Ileostomy		Total		*p*-value
	*N* = 43	3	*N* = 61	%	*N* = 104	%	
Grade of leakage (ABC)							
A	4	9.3	13	21.3	17	16.3	< 0.001
B	6	14.0	30	49.2	36	34.6	*
C	33	76.7	18	29.5	51	49.0	*
Type of leakage							
Dehiscence	27	62.8	18	29.5	45	43.3	0.002*
Abscess	11	25.6	32	52.5	43	41.3	*
Sinus	0	0.0	4	6.6	4	3.8	
Fistula	3	7.0	2	3.3	5	4.8	
Other	2	4.7	5	8.2	7	6.7	
Days until detection of leakage							
Median[IQR]	5[3–11]		12[7–32]		8[4–20.5]		< 0.001
Early or late leakage							
Diagnosis within 4 weeks	41	95.3	46	75.4	87	83.7	0.007
Diagnosis after 4 weeks	2	4.7	15	24.6	17	16.3	
Reintervention within 30 days	39	90.7	41	67.2	80	76.9	0.008
Most severe complication within 30 days (Clavien-Dindo)							
Mild grade 1–2	3	7.1	13	24.1	16	16.7	0.027
Severe Grade 3 or higher	39	92.9	41	75.9	80	83.3	
ICU admission in days							
Median[IQR]	2[1–3.5]		1[1–3]		2[1–3.25]		0.205
Admission time in days							
Median[IQR]	11[5.5–19.25]		7.5[5–17.25]		8[5–17.25]		0.384
Presence of a stoma at 1 year							
No	21	48.8	30	49.2	51	49.0	0.676
Loop ileostomy	12	27.9	19	31.1	31	29.8	
Loop colostomy	1	2.3	0	0.0	1	1.0	
End colostomy	9	20.9	12	19.7	21	20.2	

Numbers in parentheses are percentages, unless mentioned otherwise

*ICU* intensive care unit

^*^post hoc test significant for this category

The median duration between primary surgery and diagnosis of anastomotic leakage was 5[3–11] days in the non-ileostomy group and 12[7–32] days in the ileostomy group (*p *< 0.001). More late leakages after four weeks were seen in the ileostomy group (24.6% vs 4.7%, *p *= 0.007). Leakage rate at four weeks was 13.0% in the ileostomy group and 16.9% in the non-ileostomy group (*p *= 0.185). Leakage rate at one year was 15.9% in the ileostomy group and 16.8% in the non-ileostomy group (*p *= 0.540). Univariate and multivariate analysis showed no impact of ileostomy on the anastomotic leakage rate (OR 0.737(95%CI 0.460–1.180), *p *= 0.204), see supplementary Table 1. At 1 year postoperatively, the rate and type of stoma did not differ between the two groups of patients with anastomotic leakage. At 1 year, 20.9% of the patients with an anastomotic leakage in the non-ileostomy group and 19.7% of the patients with an anastomotic leakage in the ileostomy group had an end-colostomy (*p *= 0.676).

### Morbidity after stoma reversal

A total of 347 patients (87.4%) had undergone stoma reversal. In the ileostomy group, 322 out of 353 patients (91.7%) had their bowel continuity restored. In the non-ileostomy group, 25 out of 46 patients (54.3%) who had a secondary ileostomy had undergone stoma reversal. After stoma reversal, 62 patients (17.9%) had postoperative complications of which ileus was the most common complication (7.8%). Wound infection rate was 1.4%. Median time to reversal in months was longer in patients who had a secondary ileostomy compared to those who received an ileostomy during primary surgery (6[4–11] vs 3[2–4], *p *< 0.001). Thirty-four patients (9.9%) developed an incisional hernia at the previous stoma site for which 41.2% underwent surgical treatment. A new stoma was constructed after reversal in 35 cases (10.1%). The most common type of new stoma after reversal was end colostomy in 21 patients (60%). The most common reason for new stoma after reversal was anastomotic leakage at the colorectal anastomosis in 11 patients (31.4%). Table 5 shows an overview of morbidity after stoma reversal.

**Table 5:** Stoma reversal related morbidity

	Non-ileostomy*		Ileostomy		Total		*p* value
	*N* = 25	54.3%	*N* = 322	91.7%	*N* = 347	87.4%	< 0.001
Reversal related morbidity within 30 days	4	16.0	58	18.0	62	17.9	1.000
No complications	21	84.0	264	82.0	285	82.1	
Surgical complication	2	8.0	42	13.0	44	12.7	0.557
Ileus	1	4.0	26	8.1	27	7.8	0.707
Anastomotic leakage	0	0.0	6	1.9	6	1.7	1.000
Fascia dehiscence	0	0.0	0	0.0	0	0.0	
Bleeding	0	0.0	7	2.2	7	2.0	0.673
Abscess	0	0.0	1	0.3	1	0.3	1.000
Perforation	0	0.0	0	0.0	0	0.0	
Wound infection	0	0.0	5	1.6	5	1.4	1.000
Other surgical**	1	4.0	4	1.2	5	1.4	0.313
General complications	1	4.0	15	4.7	16	4.6	1.000
Pulmonary	0	0.0	1	0.3	1	0.3	1.000
Cardiac	0	0.0	3	0.9	3	0.9	1.000
Thrombotic	0	0.0	0	0.0	0	0.0	
Neurological	0	0.0	4	1.2	4	1.2	1.000
Infectious	0	0.0	3	0.9	3	0.9	1.000
Urological	0	0.0	1	0.3	1	0.3	1.000
Other	1	4.0	7	2.2	8	2.3	1.000
Other complications not specified	1	4.0	5	1.6	6	1.7	0.364
Time to reversal in months							
Median[IQR]	6[4–11]		3[2–4]		3[2–5]		< 0.001
Incisional hernia at previous stoma site							
No	21	84.0	283	88.7	304	88.4	0.523
Yes	4	16.0	30	9.4	34	9.9	
Unknown	0	0.0	6	1.9	6	1.7	
Surgical treatment of incisional hernia***							
No	1	25.0	19	63.3	20	58.8	0.231
Yes	3	75.0	11	36.7	14	41.2	
New stoma after reversal	1	4.0	34	10.6	35	10.1	0.438
Type of new stoma after reversal							
Loop ileostomy	1	100.0	2	5.9	3	8.6	0.143
End ileostomy	0	0.0	1	2.9	1	2.9	
Loop colostomy	0	0.0	9	26.5	9	25.7	
End colostomy	0	0.0	21	61.8	21	60.0	
Unknown	0	0.0	1	2.9	1	2.9	
Reason for new stoma after reversal							
Leakage after reversal	0	0.0	11	32.4	11	31.4	0.779
Poor functional outcome	0	0.0	5	14.7	5	14.3	
Palliative treatment	0	0.0	2	5.9	2	5.7	
Other	1	100.0	16	47.1	17	48.6	

Numbers in parentheses are percentages, unless mentioned otherwise

^*^ Secondary ileostomy

^**^ Others included reoperations for: 1 ileus, 1 serosa defects, 1 ileus requiring bowel resection, 1 laparoscopic lavage, 1 abscess at anastomotic site

^***^ % of patients with Incisional hernia

## Discussion

In this multicentre retrospective study, 353 patients with diverting ileostomy were compared to 242 without diverting ileostomy construction during primary TME. In the ileostomy group, 18.7% of the patients still had a stoma at one year postoperatively. In the non-ileostomy group a secondary stoma was created in 17.8% of the patients. Of these, the majority was reversed and only 9.9% of the total group had a stoma at one year postoperatively. Construction of an ileostomy at primary surgery was an independent predictor for presence of a stoma one year after surgery in a multivariate analysis. Significantly more postoperative and stoma-related morbidity was seen in the group with ileostomy construction during primary surgery. The overall rate and morbidity of anastomotic leakage was comparable between both groups, although more grade C leakages were seen in the group initially treated without ileostomy. In all the patients in the non-ileostomy group with a grade C leakage, a secondary stoma was constructed.

In most hospitals it is routine practice to construct a diverting ileostomy after a low anastomosis in rectal cancer surgery. However, a diverting ileostomy itself is related to substantial short- and long-term morbidity and therefore the advantages and disadvantages of a diverting ileostomy are debated [11, 12]. This debate results in a large variation in patient selection for diverting ileostomy construction [14]. The current data confirm that a more selective approach with proactive anastomotic leakage management might be beneficial for patients on the long-term, as was shown in previous studies [18, 19]. Routine diversion has been common practice for many years based on the idea that an ileostomy will improve the outcome of care in several ways. We would like to address several concerns that might rise at the suggestion of using a more selective approach.

First of all, critics of the selective approach suggest that the presence of an ileostomy decreases the severity of anastomotic leakage. We indeed found more severe complications (Clavien-Dindo grade 3 or higher) in the non-ileostomy group. However, the majority of these reinterventions were related to the selective diversion and active leakage management. More grade C leakage was seen in the non-ileostomy. In these cases, a secondary stoma was constructed. The more detailed subgroup analysis of patients with anastomotic leakage did not show increased severity of complications after anastomotic leakage in patients without ileostomy. Non-diverted patients do not seem to be in disadvantage in case of an anastomotic leakage. Comparable results were seen in previous studies [11, 12]. Results from a previously published Dutch cohort study showed that a high tendency towards stoma construction did not result in lower anastomotic leakage or mortality rates [14]. In accordance with the present results, Emmanuel et al. showed that although the number of reoperations after anastomotic leakage seems to be higher in patients without ileostomy, patients with an ileostomy generally require more reoperations, including planned stoma reversal [27]. With respect to the incidence of anastomotic leakage rates, the groups in the current study did not differ and anastomotic leakages rates matches those observed in previous studies [6, 28, 29]. In fact, more late leakages were seen in patients with a primary ileostomy. This is in agreement with Borstlap et al. who also showed that the diagnosis of leakage is delayed in presence of a diverting stoma [6]. Early detection and intervention for anastomotic leakage might improve the anastomotic healing rates [6, 18] and does not have an impact on oncological outcome [30]. This is more likely to succeed in absence of an ileostomy [30]. Instead of diminishing the consequences of an anastomotic leakage, delaying the diagnosis of an anastomotic leakage might actually be an important disadvantage of a diverting stoma.

Secondly, surgeons in favour of routine diversion might also claim that patients who develop anastomotic leakage might be at risk of losing the anastomosis in case of anastomotic leakage [8]. The present study however, did not show a higher anastomotic takedown rate after anastomotic leakage in the group without ileostomy. The one-year stoma rate after anastomotic leakage was comparable. In both groups about 30% end up with a loop ileostomy after anastomotic leakage, suggesting the ileostomy is not reversed after anastomotic leakage. This is in line with previous studies [10]. Interestingly, current data suggest the rate of patients with a stoma at one year was higher in the ileostomy group. Although a diverting stoma is intended to be restored, up to 20% of all patients end up with a permanent stoma. These results match those of previous studies, showing that the construction of a diverting ileostomy itself is an independent risk factor for a permanent stoma [16, 17, 31]. A logical explanation for this might be that the presence of an ileostomy is a confounder, as this group might have more advanced tumor stage and received more neoadjuvant therapy. There was a higher rate of neoadjuvant therapy administration in the ileostomy group. Indeed, neoadjuvant therapy is an independent risk factor for non-reversal of a secondary stoma [6, 15, 17]. However, in our study construction of a stoma during primary surgery was an independent risk factor for presence of a stoma at one year, even after correction for sex, age, anastomotic leakage and neoadjuvant therapy in a multivariate analysis. The increased risk of a permanent stoma after ileostomy construction during primary surgery is a clinically important problem as it exposes patients to long-term stoma related morbidity and the associated impact on quality of life [8].

Finally, other effects of a diverting ileostomy should also be considered, such as the substantial stoma-related morbidity and high readmission rates [11–13]. Postoperative morbidity rates within 30 days postoperatively were significantly higher in patients with a diverting ileostomy and stoma-related complications were present in almost half of all patients with a diverting ileostomy. Comparable results were seen in previous studies, confirming that the diverting ileostomy itself is associated with substantial morbidity, occurring in half of the patients [11, 27]. Although the grade of severity of the morbidity seemed lower in patients with an ileostomy, the overall morbidity rates were higher in this group. Stoma-related complications are known to be distressing and embarrassing for patients and cause a major burden [32]. Moreover, the general reoperation rate is higher in patients with an ileostomy, including planned stoma reversal. Stoma reversal related morbidity was 17.9%. These results match those observed in earlier studies stating that stoma reversal comes at a high risk [11, 33]. All of above can lead to increased treatment cost beyond the initial cancer treatment [13].

The current study is unique in its size and comprehensive overview of stoma-related morbidity. All diverting stomas were ileostomies to create a more homogeneous cohort. However, the study has several limitations that should be mentioned. First of all, this is a retrospective cohort study and multiple hospitals participated in this study. Therefore, different treatment protocols for anastomotic leakage were used. Moreover, like in many non-randomized studies selection bias might be apparent. There was a tendency to construct a diverting ileostomy in patients with an estimated higher risk of postoperative complications. This is reflected by a higher rate of MRI-defined low rectal cancers, cT3-4 tumours and neoadjuvant therapy in the ileostomy group. The patient- and tumour-related case-mix factors may also be responsible for a large part of the hospital variation. However, this was corrected for in a multivariate analysis, taking known confounders into account, such as: sex, comorbidity, tumour height and the administration of neoadjuvant therapy [17]. However, other confounders like individual consideration of the surgeon cannot be accounted for in this study design. Finally, some relevant data were not studied. For example, minor stoma-related morbidity such as skin irritation and plaque leakage were not registered. These complications can be distressing, are often underestimated and might require unplanned readmissions [32]. Also, data on the total length of hospital stay for the entire treatment would be interesting [34]. However, it seems very unlikely that these extra data will strengthen the conclusion of the study even more.

In conclusion, faecal diversion through diverting ileostomy after rectal cancer surgery with primary anastomosis does not reduce the anastomotic leakage rate or the morbidity caused by anastomotic leakage. On the contrary, the morbidity related to the presence and reversal of a diverting ileostomy is substantial. Furthermore, the stoma rate at one year was higher in patients who received a diverting ileostomy during primary surgery. A secondary ileostomy does not seem to hamper clinical outcomes. Future research should focus on early detection of anastomotic leakage and possible treatment options. A more selective approach to diversion could result in reduced stoma-related morbidity and stoma reversal related morbidity [18, 19]. In theory, this could lead to better quality of life and lower treatment costs [34]. Selective diversion might be safe and feasible [18, 19] and should be evaluated further, taking cost-effectivity into account.

## Supplementary Information

Below is the link to the electronic supplementary material.

Supplementary file1 (JPG 1580 kb)

Supplementary file2 (DOCX 18 kb)
