# Supplementary material for: Impact of a diverting ileostomy in total mesorectal excision with primary anastomosis for rectal cancer
Source: Surg Endosc. 2022 Oct 18;37(3):1916–32. doi: 10.1007/s00464-022-09669-x (PMC10017638; doi:10.1007/s00464-022-09669-x)
Supplement: Supplementary file 2 — Supplementary file2 (DOCX 18 kb) [file 464_2022_9669_MOESM2_ESM.docx]

**Supplementary Table 1. Outcomes of univariate logistic regression and multivariate logistic regression analyses**

| **Stoma at 1 year follow-up** | | | | |  | | | |
| --- | --- | --- | --- | --- | --- | --- | --- | --- |
| **Univariate analysis** | | | | | **Multivariate analysis** | | | |
|  | **RR** | **95%CI Lower** | **95% CI Upper** | **P-value** | **RR** | **95%CI Lower** | **95% CI Upper** | **P-value** |
| Ileostomy | 2.089 | 1.268 | 3.441 | 0.004 | 2.563 | 1.424 | 4.611 | 0.002 |
| Sex | 0.411 | 0.241 | 0.704 | 0.001 | 0.501 | 0.273 | 0.921 | 0.026 |
| Age | 1.027 | 1.002 | 1.053 | 0.034 | 1.044 | 1.012 | 1.076 | 0.006 |
| BMI | 1.041 | 0.986 | 1.100 | 0.142 |  |  |  |  |
| ASA | 1.208 | 0.848 | 1.721 | 0.295 |  |  |  |  |
| Distance ARJ on MRI | 0.907 | 0.837 | 0.983 | 0.018 |  |  |  |  |
| Neoadjuvant | 1.538 | 0.952 | 2.486 | 0.079 |  |  |  |  |
| Conversion | 0.552 | 0.156 | 1.960 | 0.358 |  |  |  |  |
| Intra-operative complication | 0.729 | 0.290 | 1.831 | 0.501 |  |  |  |  |
| cT | 1.232 | 0.852 | 1.782 | 0.267 |  |  |  |  |
| cN | 1.122 | 0.874 | 1.440 | 0.366 |  |  |  |  |
| cM | 1.112 | 0.858 | 1.443 | 0.422 |  |  |  |  |
| Leakage | 12.723 | 7.640 | 21.188 | <0.001 | 15.366 | 8.802 | 26.826 | <0.001 |

| **Overall anastomotic leakage rate** | | | | |  | | | |
| --- | --- | --- | --- | --- | --- | --- | --- | --- |
| **Univariate analysis** | | | | | **Multivariate analysis** | | | |
|  | **RR** | **95%CI Lower** | **95% CI Upper** | **P-value** | **RR** | **95%CI Lower** | **95% CI Upper** | **P-value** |
| Ileostomy | 0.962 | 0.626 | 1.479 | 0.860 | 0.737 | 0.460 | 1.180 | 0.204 |
| Sex | 0.616 | 0.387 | 0.981 | 0.041 | 0.625 | 0.389 | 1.003 | 0.052 |
| Age | 0.995 | 0.974 | 1.017 | 1.017 |  |  |  |  |
| BMI | 1.007 | 0.955 | 1.062 | 0.789 |  |  |  |  |
| ASA | 1.124 | 0.804 | 1.571 | 0.495 |  |  |  |  |
| Distance ARJ on MRI | 0.906 | 0.841 | 0.977 | 0.011 | 0.915 | 0.848 | 0.988 | 0.023 |
| Neoadjuvant | 1.578 | 1.003 | 2.484 | 0.049 | 1.598 | 0.976 | 2.615 | 0.062 |
| Conversion | 0.679 | 0.241 | 1.919 | 0.466 |  |  |  |  |
| Intra-operative complication | 1.109 | 0.416 | 2.960 | 0.836 |  |  |  |  |

| **Complications within 30 days** | | | | |  | | | |
| --- | --- | --- | --- | --- | --- | --- | --- | --- |
| **Univariate analysis** | | | | | **Multivariate analysis** | | | |
|  | **RR** | **95%CI Lower** | **95% CI Upper** | **P-value** | **RR** | **95%CI Lower** | **95% CI Upper** | **P-value** |
| Ileostomy | 2.120 | 1.517 | 2.961 | <0.001 | 2.037 | 1.434 | 2.892 | <0.001 |
| Sex | 0.375 | 0.265 | 0.531 | <0.001 | 0.398 | 0.278 | 0.569 | <0.001 |
| Age | 1.006 | 0.989 | 1.023 | 0.491 |  |  |  |  |
| BMI | 1.013 | 0.973 | 1.055 | 0.527 |  |  |  |  |
| ASA | 1.516 | 1.167 | 1.968 | 0.002 | 1.550 | 1.175 | 2.045 | 0.002 |
| Distance ARJ on MRI | 0.966 | 0.913 | 1.023 | 0.238 |  |  |  |  |
| Neoadjuvant | 1.492 | 1.071 | 2.078 | 0.018 |  |  |  |  |
| Conversion | 1.364 | 0.709 | 2.622 | 0.353 |  |  |  |  |
| Intra-operative complication | 0.767 | 0.371 | 1.586 | 0.474 |  |  |  |  |

| **Anastomotic leakage grade C** | | | | |  | | | |
| --- | --- | --- | --- | --- | --- | --- | --- | --- |
| **Univariate analysis** | | | | | **Multivariate analysis PSA*** | | | |
|  | **RR** | **95%CI Lower** | **95% CI Upper** | **P-value** | **RR** | **95%CI Lower** | **95% CI Upper** | **P-value** |
| Ileostomy | 0.340 | 0.187 | 0.620 | <0.001 | 0.263 | 0.138 | 0.505 | <0.001 |

* PSA: propensity score adjusted analysis. Adjusted for: sex, age, BMI, ASA, distance from anorectal junction on MRI, neoadjuvant treatment, conversion, intra-operative complication
